# Association Between the Change in Interleukin‐6 Levels and Early Recurrence of Atrial Fibrillation: A Comparison of Pulsed Field Ablation and Radiofrequency Ablation

**DOI:** 10.1002/joa3.70456

**Published:** 2026-09-17

**Authors:** Ken Watanabe, Takanori Arimoto, Mashu Toyoshima, Kyoko Koyama, Yuta Kobayashi, Naoaki Hashimoto, Masafumi Watanabe

**Affiliations:** ^1^ Department of Cardiology, Pulmonology, and Nephrology Yamagata University School of Medicine Yamagata Japan

**Keywords:** early recurrence of atrial fibrillation, interleukin‐6, pulsed field ablation

## Abstract

**Background:**

Early recurrence of atrial fibrillation (ERAF) within the 3‐month blanking period is associated with procedural inflammation and increased risk of late recurrence. Pulsed field ablation (PFA) reportedly has a lower ERAF rate than radiofrequency ablation (RFA), but the underlying inflammatory mechanisms remain unclear.

**Methods:**

We compared 23 patients undergoing atrial fibrillation ablation (PFA: *n* = 16; RFA: *n* = 7). Perioperative changes in inflammatory markers, myocardial injury markers, hemolysis markers, and the incidence of ERAF were analyzed.

**Results:**

PFA exhibited significantly greater myocardial injury and hemolysis, but markedly lower systemic inflammation than RFA, characterized by lower Δinterleukin‐6 (IL‐6) levels (4.6 ± 1.7 vs. 8.1 ± 3.8 pg/mL, *p* = 0.008). ERAF occurred more frequently in RFA (3/7, 43% vs. 1/16, 6%, *p* = 0.033), which was accompanied by significantly higher ΔIL‐6 levels (9.1 ± 4.2 vs. 4.9 ± 2.3 pg/mL, *p* = 0.010). Multivariate logistic regression analysis identified ΔIL‐6 as an independent predictor of ERAF (OR 2.158, 95% CI 1.125–13.020, *p* = 0.013).

**Conclusions:**

PFA induces less systemic inflammation than RFA, despite causing greater myocardial injury. This reduced inflammatory response is independently associated with the lower incidence of ERAF.

## Graphical abstract

Despite causing greater myocardial injury and hemolysis, pulsed field ablation induces significantly less systemic inflammation than radiofrequency ablation. This reduced inflammatory response is independently associated with a lower incidence of early atrial fibrillation recurrence.

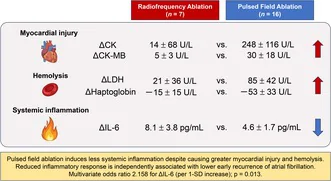

## 1 Introduction

Catheter ablation is the established treatment for drug‐refractory atrial fibrillation (AF). Inflammation has been closely associated with the risk of AF recurrence, and anti‐inflammatory therapies have been reported to reduce recurrence of AF after catheter ablation [1, 2]. In particular, procedural inflammation has been identified as a key driver of early recurrence of atrial fibrillation (ERAF) within the 3‐month blanking period, which is a strong predictor of late recurrence [3, 4]. While thermal injury from radiofrequency ablation (RFA) is known to trigger a robust systemic inflammatory response, pulsed field ablation (PFA) has emerged as a non‐thermal modality that utilizes irreversible electroporation to ablate myocardial tissue preferentially [5]. Recent reports suggest that PFA is associated with lower ERAF rates compared to RFA [6, 7]. However, the pathophysiological mechanisms driving this difference are not fully understood. The purpose of this study was to compare perioperative changes in markers of inflammation, myocardial injury, and hemolysis between PFA and RFA. Furthermore, we sought to determine the association between these biomarkers and the incidence of ERAF.

## 2 Materials and Methods

### 2.1 Study Subjects

The present study enrolled 23 patients (the RFA group: *n* = 7; the PFA group: *n* = 16) who underwent their first catheter ablation for AF at Yamagata University Hospital between May and October 2025. The choice of the ablation modality was determined at the discretion of the attending physician. RFA was preferentially selected for patients with a history of asthma who could not receive thiopental for deep sedation, and for patients with concomitant typical atrial flutter scheduled for cavotricuspid isthmus (CTI) ablation. The exclusion criteria were active hepatic disease, pulmonary disease, malignant disease, systemic inflammatory disease, and history of open‐heart surgery. This study was conducted in accordance with the principles outlined in the Declaration of Helsinki. The study protocol was approved by the institutional ethics committee of Yamagata University School of Medicine (Approval No. 2026‐95) and all patients provided their written informed consent.

### 2.2 Ablation Procedures

In the RFA group, high‐power short‐duration radiofrequency pulmonary vein isolation (PVI) was performed using contact‐force sensing catheters. In the PFA group, PVI was performed using one of the commercially available PFA systems: a pentaspline catheter (FARAPULSE, Boston Scientific, *n* = 6) or 2 loop circular catheters (VARIPULSE, Biosense Webster, *n* = 6; and PulseSelect, Medtronic, *n* = 4). Patients in the RFA group underwent the procedure under mild sedation using a combination of dexmedetomidine and fentanyl. In contrast, patients in the PFA group received deep sedation using a combination of dexmedetomidine, midazolam, thiopental, and fentanyl. Additional ablations, such as cavotricuspid isthmus (CTI) ablation, superior vena cava isolation, and posterior wall isolation, were performed at the operator's discretion.

Each PFA system was delivered into the left atrium via its dedicated deflectable sheath (FARADRIVE for FARAPULSE, VIZIGO for VARIPULSE, or FlexCath Contour for PulseSelect). The FARAPULSE was performed with 4 applications in the basket configuration and 4 in the flower configuration per PV. One application consisted of 5 consecutive pulse energies. The VARIPULSE was performed with 2 applications targeting the PV ostium and 2 targeting the antrum. One application consisted of 3 consecutive pulse energies. The PulseSelect was performed with at least 4 applications at the PV ostium and the antrum. One application consisted of 4 consecutive pulse energies. Additional PFA was delivered if PV gaps were identified. The procedural end point was defined as the completion of PVI.

### 2.3 Biomarker Analysis

Blood samples were collected at three specified time points to evaluate perioperative changes: baseline at admission, immediately after ablation, and at approximately 9:00 AM on the day after ablation. We analyzed systemic inflammation markers, including white blood cells (WBC), C‐reactive protein (CRP), and interleukin‐6 (IL‐6). Additionally, markers of myocardial injury (creatine kinase [CK], CK‐MB, and troponin T) and hemolysis (lactate dehydrogenase [LDH] and haptoglobin) were evaluated. The change (Δ) in these parameters from baseline to the morning after ablation was calculated for analysis, except for IL‐6 which was evaluated at three time points. The samples were centrifuged at 3000 g for 15 min at 4°C, and the obtained serum was stored at −80°C until analysis. Serum IL‐6 levels were measured with a sandwich enzyme‐linked immunosorbent assay (ELISA, R&D systems Inc., Minneapolis, MN, USA, Cat #HS600C) according to the manufacturer's instructions.

### 2.4 Endpoint and Follow‐Up Period

Assessments of AF recurrence were performed according to our institutional protocol. Clinical follow‐up visits were conducted at 1 and 3 months after the ablation procedure. Routine arrhythmia monitoring included 12‐lead electrocardiogram (ECG) at every clinic visit. In addition, symptom‐driven self‐monitoring was implemented, using devices such as portable ECG monitors and smartwatches. All event recordings were assessed by experienced cardiac electrophysiologists. The primary outcome of this study was the incidence of ERAF, which is defined as any documented episode of AF lasting > 30 s that occurred within the 3‐month blanking period after the ablation procedure.

### 2.5 Statistical Analysis

Continuous variables are expressed as mean ± standard deviation (SD) or median and interquartile range (IQR) depending on their distribution. Categorical variables are presented as numbers and percentages. Comparisons between the RFA group and the PFA group were performed using *t*‐tests and chi‐square tests to compare continuous and categorical variables, respectively. If the data were not normally distributed, the Mann–Whitney *U*‐test was employed. Differences among groups were analyzed by one‐way analysis of variance. Univariate linear regression analysis was performed to identify factors associated with increased IL‐6 levels, and the results were expressed as standardized coefficients (*β*). The analysis evaluating the number of PFA applications included only the 16 patients in the PFA group. Univariate and multivariate logistic regression analyses were performed to identify independent predictors of ERAF. *p*‐values of < 0.05 were considered statistically significant. All statistical analyses were performed with a standard software package (JMP version 18, SAS Institute, Cary, NC, USA).

## 3 Results

### 3.1 Patient and Procedural Characteristics

The baseline patient characteristics of the study population are summarized in Table 1. There were no significant differences in age, body mass index, CHADS_2_/CHA_2_DS_2_‐VASc scores, AF type, or the prevalence of comorbidities such as hypertension, diabetes mellitus, and heart failure between the RFA and the PFA groups. Although not statistically significant, there was a trend toward a higher proportion of male patients, a lower left ventricular ejection fraction, and a more frequent use of β‐blockers in the RFA group compared to the PFA group. All patients in both groups were receiving oral anticoagulants at baseline.

**TABLE 1:** Patient characteristics.

Variables	RFA group *n* = 7	PFA group *n* = 16	*p*
Age (years)	65 ± 11	64 ± 12	0.749
Male sex, *n* (%)	7 (100)	11 (69)	0.095
BMI (kg/m^2^)	23.4 ± 1.2	25.1 ± 3.7	0.250
Hypertension, *n* (%)	4 (57)	12 (75)	0.392
Diabetes mellitus, *n* (%)	1 (14)	2 (13)	0.907
Heart failure, *n* (%)	2 (28)	2 (13)	0.349
Smoking, *n* (%)	1 (14)	3 (19)	0.792
CHADS_2_ score	1.1 ± 1.3	1.1 ± 0.7	0.967
CHA_2_DS_2_‐VASc score	1.7 ± 1.7	1.9 ± 1.1	0.712
AF type			
Paroxysmal/Persistent	6/1	12/4	0.567
Echocardiographic data			
LAD (mm)	39 ± 4	43 ± 7	0.194
LAVI (mL/m^2^)	35 ± 15	40 ± 15	0.508
LVEF (%)	57 ± 16	67 ± 10	0.073
Blood examination			
eGFR (mL/min/1.73 m^2^)	62.0 ± 16.3	68.4 ± 14.8	0.360
Hemoglobin (g/dL)	14.9 ± 1.3	14.2 ± 1.7	0.355
BNP (pg/mL)	46 (14–143)	20 (13–70)	0.423
Medications			
OAC, *n* (%)	7 (100)	16 (100)	1.000
β‐blockers, *n* (%)	6 (86)	7 (44)	0.062
Class I/III AAD, *n* (%)	4 (57)	8 (50)	0.752
Statins, *n* (%)	1 (14)	5 (31)	0.375

*Note:* Data are expressed as mean ± SD, number (percentage), or median (interquartile range).

Abbreviations: AAD, antiarrhythmic drug; AF, atrial fibrillation; BMI, body mass index; BNP, B‐type natriuretic peptide; eGFR, estimated glomerular filtration rate; LAD, left atrial diameter; LAVI, left atrial volume index; LVEF, left ventricular ejection fraction; OAC, oral anticoagulant; PFA, pulsed field ablation; RFA, radiofrequency ablation.

The procedural characteristics are shown in Table S1. The procedural duration was significantly shorter in the PFA group compared to the RFA group (90 ± 29 vs. 146 ± 38 min, *p* < 0.001). Similarly, left atrial dwell time was shorter in the PFA group (64 ± 25 vs. 113 ± 26 min, *p* < 0.001). Additional CTI ablation was more frequent in the RFA group (57% vs. 0%, *p* < 0.001).

### 3.2 Myocardial Injury and Hemolysis

Perioperative parameter changes from baseline to the morning following ablation are summarized in Table 2. The PFA group exhibited significantly higher elevations in myocardial injury markers compared to the RFA group, as evidenced by greater increases in ΔCK (248 ± 116 vs. 14 ± 68 U/L, *p* < 0.001) and ΔCK‐MB (30 ± 18 vs. 5 ± 3 U/L, *p* = 0.001). Furthermore, markers of hemolysis were more prominent in the PFA group, characterized by a significantly higher ΔLDH (85 ± 42 vs. 21 ± 36 U/L, *p* = 0.002) and a greater decline in Δhaptoglobin (−53 ± 33 vs. −15 ± 15 mg/dL, *p* = 0.015). There was no significant difference in Δtroponin T between the two groups. Regarding renal function, ΔeGFR showed a slight decrease in the PFA group (−2.1 ± 5.1 vs. 5.0 ± 10.1 mL/min/1.73 m^2^, *p* = 0.033).

**TABLE 2:** Changes in parameters before and after ablation.

Variables	RFA group *n* = 7	PFA group *n* = 16	*p*
ΔSystolic blood pressure (mmHg)	−7 ± 23	−5 ± 19	0.777
ΔDiastolic blood pressure (mmHg)	−4 ± 11	−7 ± 13	0.607
ΔHeart rate (bpm)	−1 ± 8	−7 ± 13	0.343
ΔBody temperature (°C)	0.9 ± 0.8	0.0 ± 0.4	0.001
Blood examination			
ΔeGFR (mL/min/1.73 m^2^)	5.0 ± 10.1	−2.1 ± 5.1	0.033
ΔCK (U/L)	14 ± 68	248 ± 116	< 0.001
ΔCK‐MB (U/L)	5 ± 3	30 ± 18	0.001
ΔTotal bilirubin (mg/dL)	0.4 ± 0.3	0.6 ± 0.5	0.494
ΔLDH (U/L)	21 ± 36	85 ± 42	0.002
ΔTroponin T (ng/mL)	1.5 (1.3–1.8)	1.6 (1.4–1.9)	0.765
ΔHaptoglobin (mg/dL)	−15 ± 15	−53 ± 33	0.015
ΔWBC (×10^3^/μL)	2.0 ± 1.8	1.5 ± 1.0	0.376
ΔCRP (mg/dL)	0.65 (0.01–0.86)	0.37 (0.21–0.55)	0.442

*Note:* Data are expressed as mean ± SD, number (percentage), or median (interquartile range).

Abbreviations: bpm, beats per minute; CK, creatine kinase; CRP, C‐reactive protein; eGFR, estimated glomerular filtration rate; LDH, lactate dehydrogenase; PFA, pulsed field ablation; RFA, radiofrequency ablation; WBC, white blood cell.

### 3.3 Systemic Inflammatory Response

**FIGURE 1:**
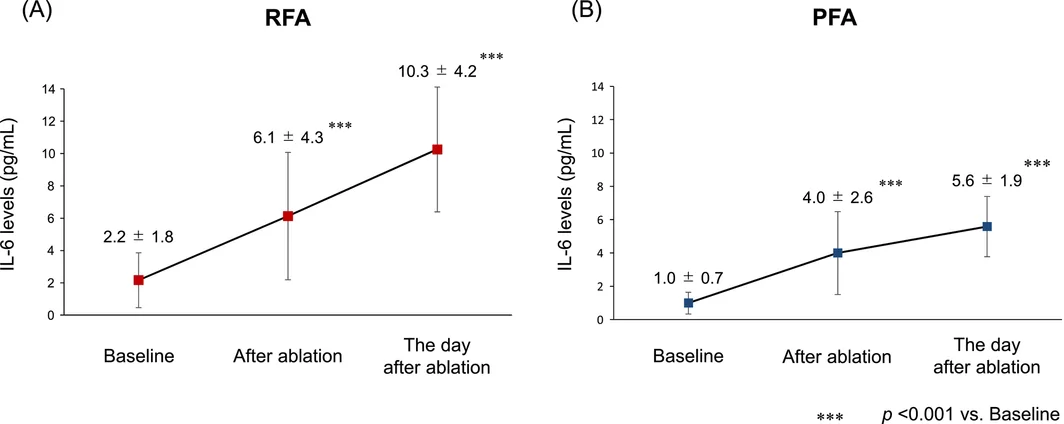
Time course of serum IL‐6 levels before and after ablation. Line graphs illustrating the serial changes in serum IL‐6 levels in the RFA (A) and the PFA (B). Blood samples were collected at baseline, immediately after ablation, and the day after ablation. In both groups, IL‐6 levels increased significantly from baseline to the post‐procedural time points (*p* < 0.001 vs. Baseline). Data are presented as individual plots with mean ± standard deviation. IL‐6, interleukin‐6; PFA, pulsed field ablation; RFA, radiofrequency ablation.

**FIGURE 2:**
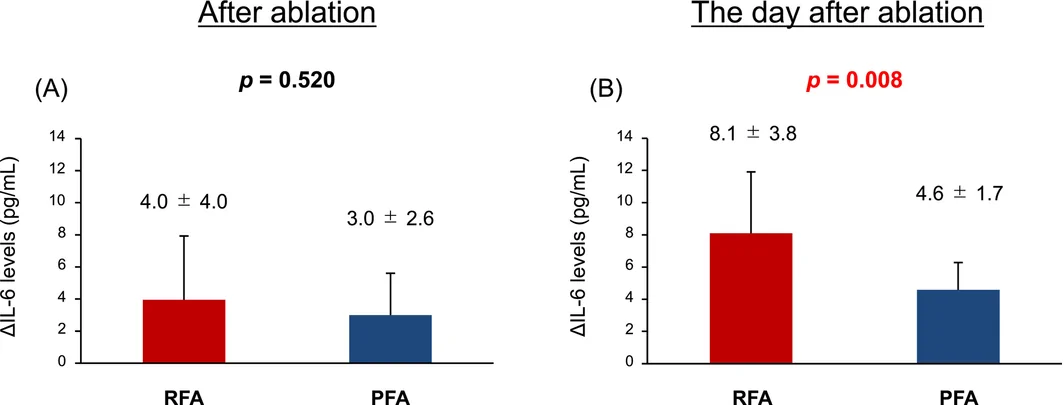
Comparison of the change in IL‐6 levels between the RFA and the PFA groups. Bar graphs comparing the changes in serum IL‐6 levels from baseline. (A) There was no significant difference in ΔIL‐6 immediately after ablation. (B) The elevation of IL‐6 levels was significantly attenuated in the PFA group compared to the RFA group. Data are expressed as mean ± standard deviation. IL‐6, interleukin‐6; PFA, pulsed field ablation; RFA, radiofrequency ablation.

Despite the significantly greater extent of myocardial injury and hemolysis, the systemic inflammatory response was paradoxically blunted in patients treated with PFA. A significantly greater post‐procedural increase in body temperature was observed in the RFA group compared to the PFA group (0.9 ± 0.8 vs. 0.0 ± 0.4, *p* = 0.001). While the changes in inflammatory markers such as ΔWBC and ΔCRP did not reach statistical significance between the groups, the kinetics of IL‐6 differed markedly. As shown in Figure 1, serum IL‐6 levels increased significantly from baseline at both immediately after ablation and the day after ablation in both the RFA and the PFA group, respectively (*p* < 0.001 vs. baseline for all). While the ΔIL‐6 levels were comparable between the groups immediately after ablation, the elevation was significantly higher in the RFA group than in the PFA group on the following day (8.1 ± 3.8 vs. 4.6 ± 1.7 pg/mL, *p* = 0.008; Figure 2).

### 3.4 Predictors of Early Recurrence of Atrial Fibrillation

**FIGURE 3:**
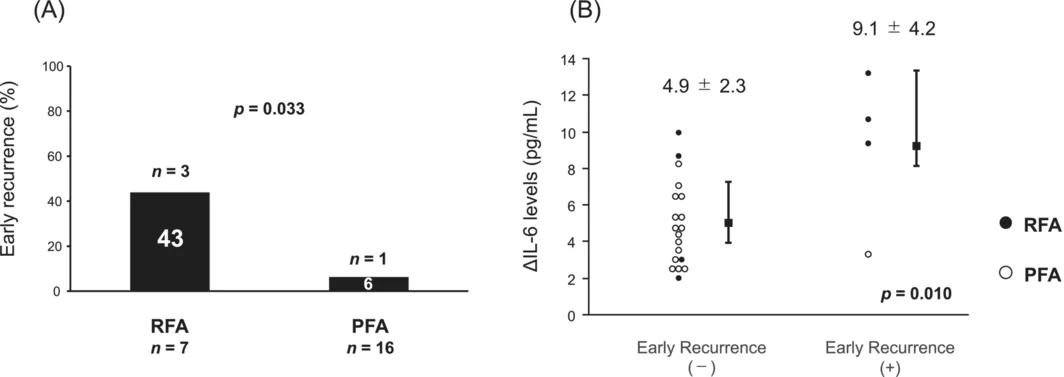
Association between the change in IL‐6 levels and ERAF. (A) The incidence of ERAF was observed significantly more frequently in the RFA group than in the PFA group. (B) Patients who experienced ERAF exhibited significantly higher ΔIL‐6 levels compared to those without ERAF. Individual patient data points are shown, with colors indicating the ablation modality. ERAF, early recurrence of atrial fibrillation; IL‐6, interleukin‐6; PFA, pulsed field ablation; RFA, radiofrequency ablation.

The ERAF occurred significantly more frequently in the RFA group than in the PFA group (3/7, 43% vs. 1/16, 6%, *p* = 0.033; Figure 3A). Patients who experienced ERAF exhibited significantly elevated ΔIL‐6 levels compared to those without ER (9.1 ± 4.2 vs. 4.9 ± 2.3 pg/mL, *p* = 0.010; Figure 3B). Univariate and multivariate logistic regression analyses for predicting the ERAF were shown in Table 3. The univariate analysis showed that a greater change in IL‐6 levels from baseline (ΔIL‐6) was significantly associated with an increased risk of ERAF (Odds ratio [OR] 1.633, 95% confidence interval [CI] 1.099–3.003, *p* = 0.013). Although not statistically significant, there were trends for smaller increase of CK levels (*p* = 0.058) and lower BMI (*p* = 0.094). The multivariate analysis revealed that elevated ΔIL‐6 levels were an independent predictor of ERAF after adjustment for confounding factors (OR 2.158, 95% CI 1.125–13.020, *p* = 0.013). Additionally, lower BMI was independently associated with an increased risk of ERAF in this study (OR 0.223, 95% CI 0.007–0.889, *p* = 0.026).

**TABLE 3:** Univariate and multivariate logistic regression analyses for predicting the early recurrence of atrial fibrillation.

Variables	Univariate analysis			Multivariate analysis		
	OR	95% CI	*p*	OR	95% CI	*p*
Age^a^	0.993	0.906–1.113	0.880			
Male sex	0.800	0.075–18.590	0.864			
BMI^a^	0.649	0.287–1.058	0.094	0.223	0.007–0.889	0.026
Hypertension	1.385	0.141–31.250	0.792			
Diabetes mellitus	2.833	0.111–41.190	0.468			
Heart failure	1.778	0.074–20.930	0.671			
Smoking	1.778	0.074–20.930	0.671			
CHADS_2_ score^a^	1.182	0.306–3.537	0.775			
CHA_2_DS_2_‐VASc score^a^	0.911	0.335–2.114	0.833			
Persistent AF	5.333	0.489–62.970	0.161			
Statins use	0.933	0.041–9.491	0.956			
LAD^a^	0.987	0.818–1.172	0.877			
LAVI^a^	1.012	0.931–1.086	0.756			
LVEF^a^	0.972	0.897–1.051	0.456			
ΔCK^a^	0.991	0.978–1.001	0.058	0.996	0.977–1.006	0.454
ΔCK‐MB^a^	0.934	0.816–1.015	0.127			
ΔTroponin T^a^	0.301	0.006–3.917	0.403			
ΔHaptoglobin^a^	0.992	0.941–1.022	0.655			
ΔIL‐6^a^	1.633	1.099–3.003	0.013	2.158	1.125–13.020	0.013

Abbreviations: AF, atrial fibrillation; BMI, body mass index; CI, confidence interval; CK, creatine kinase; IL‐6, interleukin‐6; LAD, left atrial diameter; LAVI, left atrial volume index; LVEF, left ventricular ejection fraction; OR, odds ratio.

^a^ Per 1‐SD increase.

**FIGURE 4:**
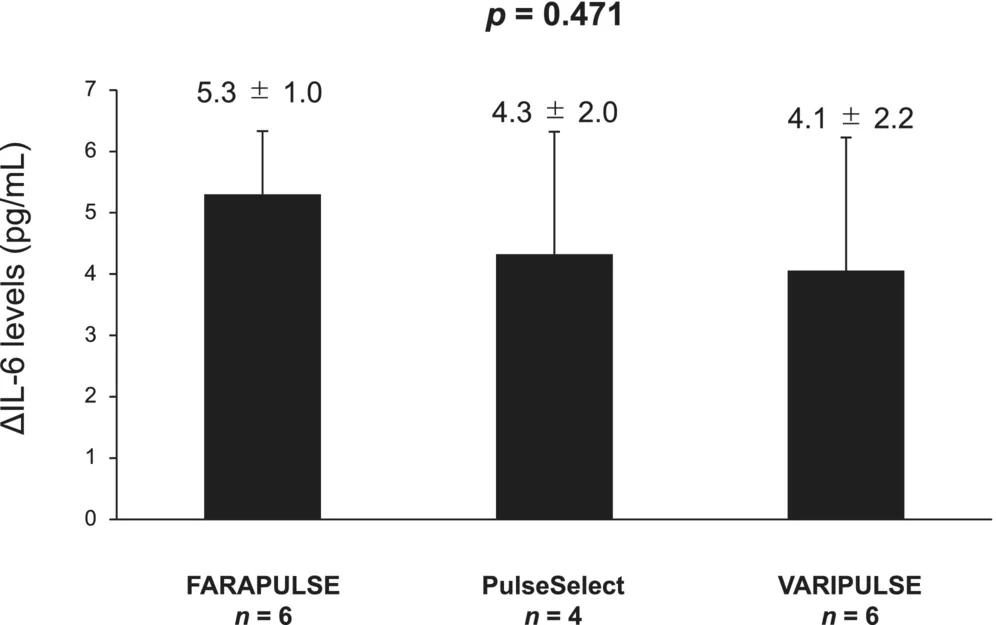
Comparison of the change in IL‐6 levels among the three PFA systems. Bar graph demonstrating the change in IL‐6 levels on the day after ablation across the three different commercially available PFA systems evaluated in this study: FARAPULSE (*n* = 6), PulseSelect (*n* = 4), and VARIPULSE (*n* = 6). There were no significant differences in the attenuated inflammatory response among the three systems. Data are expressed as mean ± standard deviation. IL‐6, interleukin‐6; PFA, pulsed field ablation.

Linear regression analysis was performed to identify factors associated with post‐procedural IL‐6 elevation (Table S2). The use of PFA was inversely associated with ΔIL‐6 levels (Standardized coefficient (*β*) = −0.536, *p* = 0.008). Significant positive correlations were found with procedural duration (*β* = 0.453, *p* = 0.030) and left atrial dwell time (*β* = 0.414, *p* = 0.049), while showing a negative correlation with Δsystolic blood pressure (*β* = −0.426, *p* = 0.043). No significant associations were observed between the elevation of IL‐6 and parameters of myocardial injury and hemolysis, including ΔCK (*p* = 0.425), ΔCK‐MB (*p* = 0.458), Δtroponin T (*p* = 0.478), ΔLDH (*p* = 0.732), and Δhaptoglobin (*p* = 0.670). Furthermore, the number of PFA applications did not correlate significantly with ΔIL‐6 levels. As shown in Figure 4, comparisons of the three PFA systems revealed no significant differences in ΔIL‐6 increments (FARAPULSE: 5.3 ± 1.0 pg/mL; PulseSelect: 4.3 ± 2.0 pg/mL; VARIPULSE: 4.1 ± 2.2 pg/mL; *p* = 0.471).

## 4 Discussion

### 4.1 Main Findings

The main findings of the present study were as follows: (i) PFA resulted in greater myocardial injury marker elevation and significant hemolysis, yet paradoxically induced a markedly blunted systemic inflammatory response, as evidenced by significantly lower ΔIL‐6 levels; (ii) PFA was associated with a significantly lower incidence of ERAF compared to RFA. Crucially, multivariate analysis revealed that the elevation of IL‐6 levels was an independent predictor of ERAF.

### 4.2 Markers of Inflammation, Myocardial Injury, and Hemolysis

RFA relies on thermal energy, which typically causes coagulative necrosis, severe tissue edema, and a robust systemic inflammatory cascade [8, 9]. In contrast, PFA utilizes high‐voltage electrical fields to induce irreversible electroporation. This electroporation‐induced cell death involves non‐thermally driven necrosis, leading to a less inflammatory response than the coagulative necrosis caused by the thermal energy ablation [10]. Consequently, patients treated with PFA exhibited a paradoxically blunted systemic inflammatory response, with negligible fever and significantly lower ΔIL‐6 levels, despite the extensive myocardial injury and hemolysis. Intriguingly, changes in these biomarkers were consistent across the three PFA systems (FARAPULSE, PulseSelect, and VARIPULSE). This uniformity implies that the suppression of systemic inflammation is a class effect of non‐thermal electroporation therapy, independent of specific catheter type.

### 4.3 Impact of Serum Interleukin‐6 Levels on Early Recurrence of Atrial Fibrillation

ERAF after AF ablation within the blanking period is a strong predictor of late recurrence [11]. Late recurrence of AF remains a clinical challenge, as it impairs patient quality of life, necessitates the prolonged use of antiarrhythmic drugs, and leads to a substantial increase in overall healthcare costs [12, 13]. Recent comparative studies have demonstrated that PFA was associated with a lower incidence of ERAF during the blanking period compared to thermal energy ablation, such as RFA and cryoballoon ablation [14, 15].

The pathophysiological mechanisms underlying ERAF are multifactorial, with post‐procedural inflammation and autonomic nervous system imbalance recognized as key contributors [16, 17]. Thermal energy ablation induces inflammatory responses, which are well‐established triggers of arrhythmogenic foci during the blanking period. In addition, RF energy delivery around the PVs can cause autonomic dysfunction and excessive sympathetic activation, further promoting ERAF. PFA, however, has been reported to elicit a significantly attenuated acute inflammatory response than conventional thermal energy sources [18, 19]. Consistent with previous studies, the present study demonstrated that PFA was associated with a lower rate of ERAF and a blunted inflammatory response compared with RFA, as evidenced by the attenuated elevation of serum IL‐6 levels. Although the pathophysiological mechanisms underlying the lower rate of ERAF in PFA remain unclear, the attenuated inflammatory response may be one of the contributing factors. Recent large‐scale trials have indicated that 1‐year recurrence rates of AF were comparable between PFA and thermal ablation [20, 21]. On the other hand, the SINGLE SHOT CHAMPION trial, which utilized continuous monitoring with an implantable loop recorder, provided critical insights into the acute healing phase [14]. The trial demonstrated that the burden of atrial arrhythmia during the blanking period was significantly lower with PFA compared to cryoballoon ablation, whereas the rates of late recurrence (days 91–365) were similar. Our results aligned with this reduced rate of ERAF in PFA, suggesting that the non‐thermal irreversible electroporation, characterized by attenuated IL‐6 elevation, can specifically protect against inflammation‐driven arrhythmias during this highly vulnerable phase.

## 5 Limitations

This study has several limitations. First, it was a single‐center, non‐randomized observational study with a relatively small sample size, particularly in the RFA group, which may introduce selection bias. Second, there was a trend toward differences in baseline characteristics, such as a higher proportion of males and a lower baseline left ventricular ejection fraction in the RFA group. Additionally, the baseline IL‐6 levels were significantly higher in the RFA group compared to the PFA group (2.2 ± 1.8 vs. 1.0 ± 0.7 pg/mL, *p* = 0.033). Although not statistically significant, IL‐6 levels tended to be higher in males than in females in our cohort (1.5 ± 0.3 vs. 1.0 ± 0.6 pg/mL, *p* = 0.437). This higher baseline IL‐6 in the RFA group may be driven by the higher proportion of male patients and heart failure cases. Third, the sample sizes within the individual PFA system subgroups were too small to definitively rule out minor differences in inflammatory profiles between specific devices. Fourth, additional CTI ablation was performed more frequently in the RFA group because PFA systems were not utilized for CTI ablation in this study. However, a sub‐analysis within the RFA group revealed no significant differences in ΔIL‐6 levels between patients with and without concomitant CTI ablation (6.7 ± 5.2 vs. 10.0 ± 0.7 pg/mL, *p* = 0.338). Finally, our study primarily focused on the acute and subacute phases; larger studies with extended follow‐up are needed to determine whether the reduction in ERAF specifically driven by low inflammation translates to superior long‐term arrhythmia‐free survival.

## 6 Conclusions

PFA induces significantly less systemic inflammation, characterized by attenuated IL‐6 elevation, compared to RFA, despite causing greater myocardial injury and hemolysis. This reduced inflammatory response is independently associated with a lower incidence of ERAF after AF ablation. These findings suggest that the non‐thermal irreversible electroporation provides a more favorable acute healing profile, contributing to improved early clinical outcomes during the blanking period.

## Funding

The authors have nothing to report.

## Ethics Statement

The study protocol was approved by the institutional ethics committee of Yamagata University School of Medicine (Approval No. 2026‐95).

## Conflicts of Interest

The authors declare no conflicts of interest.

## Supporting information


**Table S1:** Procedural characteristics.
**Table S2:** Factors associated with increased IL‐6 levels.

## Data Availability

The data that support the findings of this study are available on request from the corresponding author. The data are not publicly available due to privacy or ethical restrictions.
