# Supplementary material for: Association Between the Change in Interleukin‐6 Levels and Early Recurrence of Atrial Fibrillation: A Comparison of Pulsed Field Ablation and Radiofrequency Ablation
Source: J Arrhythm. 2026 Sep 17;42(5):e70456. doi: 10.1002/joa3.70456 (PMC13586436; doi:10.1002/joa3.70456)
Supplement: Supplementary file 1 [file JOA3-42-e70456-s001.docx]

**Table S1.** Procedural characteristics

| Variables | RFA group  n = 7 | PFA group  n = 16 | *P* value |
| --- | --- | --- | --- |
| Procedural duration (min) | 146 ± 38 | 90 ± 29 | <0.001 |
| LA dwell time (min) | 113 ± 26 | 64 ± 25 | <0.001 |
| Fluoroscopy duration (min) | 10 ± 9 | 21 ± 10 | 0.021 |
| Fluoroscopy dose (mGy) | 65 ± 63 | 160 ± 125 | 0.074 |
| Additional ablation |  |  |  |
| CTI ablation, n (%) | 4 (57) | 0 (0) | <0.001 |
| SVC isolation, n (%) | 1 (14) | 0 (0) | 0.122 |
| Posterior LA wall isolation, n (%) | 0 (0) | 1 (6) | 0.499 |
| Number of PFA applications | n/a | 49 ± 11 | n/a |
| Complications, n (%) | 0 (0) | 0 (0) | 1.000 |

Data are expressed as mean ± SD, number (percentage).

CTI, cavotricuspid isthmus; LA, left atrium; n/a, not applicable; PFA, pulsed field ablation; RFA, radiofrequency ablation, SVC, superior vena cava.

**Table S2.** Factors associated with increased IL-6 levels

|  | *IL6* | | |
| --- | --- | --- | --- |
|  | Standardized Coefficient (β) | R^2^ | *P* value |
| Age | 0.133 | 0.018 | 0.545 |
| Male sex | 0.398 | 0.159 | 0.060 |
| BMI | －0.057 | 0.003 | 0.795 |
| Hypertension | 0.084 | 0.007 | 0.703 |
| Diabetes mellitus | 0.056 | 0.003 | 0.799 |
| Heart failure | 0.239 | 0.057 | 0.272 |
| Smoking | －0.021 | <0.001 | 0.926 |
| CHADS_2_ score | 0.198 | 0.039 | 0.365 |
| CHA_2_DS_2_-VASc score | 0.038 | 0.001 | 0.865 |
| Persistent AF | 0.011 | <0.001 | 0.960 |
| PFA | －0.536 | 0.287 | 0.008 |
| Procedural duration | 0.453 | 0.206 | 0.030 |
| LA dwell time | 0.414 | 0.172 | 0.049 |
| Fluoroscopy duration | －0.344 | 0.118 | 0.108 |
| Fluoroscopy dose | 0.206 | 0.042 | 0.346 |
| Number of PFA applications | －0.319 | 0.102 | 0.228 |
| ΔSystolic blood pressure | －0.426 | 0.181 | 0.043 |
| ΔDiastolic blood pressure | －0.237 | 0.056 | 0.276 |
| ΔHeart rate | 0.090 | 0.008 | 0.684 |
| ΔBody temperature | 0.057 | 0.033 | 0.408 |
| ΔeGFR | －0.148 | 0.022 | 0.502 |
| ΔCK | －0.244 | 0.060 | 0.425 |
| ΔCK-MB | 0.026 | 0.026 | 0.458 |
| ΔTotal bilirubin | 0.128 | 0.016 | 0.562 |
| ΔLDH | －0.075 | 0.006 | 0.732 |
| ΔTroponin T | 0.164 | 0.027 | 0.478 |
| ΔHaptoglobin | 0.096 | 0.009 | 0.670 |
| ΔWBC | 0.219 | 0.048 | 0.316 |
| ΔCRP | 0.345 | 0.119 | 0.107 |

AF, atrial fibrillation; BMI, body mass index; CK, creatine kinase; CRP, C-reactive protein; eGFR, estimated glomerular filtration rate; IL-6, interleukin-6; LA, left atrium; LDH, lactate dehydrogenase; PFA, pulsed field ablation; WBC, white blood cell.
